# Splenic and portal vein thrombosis in pancreatic metastasis from Renal cell carcinoma

**DOI:** 10.1186/1477-7819-4-25

**Published:** 2006-05-17

**Authors:** Shailesh V Shrikhande, Peter Büchler, Irene Esposito, Martin Loos, Markus W Büchler, Helmut Friess

**Affiliations:** 1 Department of General Surgery, University of Heidelberg, Heidelberg, Germany; 2 Department of Pathology, University of Heidelberg, Heidelberg, Germany

## Abstract

**Background:**

Pancreatic metastases from previously treated renal cell carcinoma are uncommon. Surgical resection of pancreatic metastasis remains the only worthwhile modality of treatment.

**Case presentation:**

A case where pancreatic metastasis from previously resected right sided renal cell carcinoma was resected with a subtotal left pancreatectomy is described. An unusual feature was the presence of a large splenic vein tumor thrombus extending into the portal vein with associated portal hypertension. The patient underwent an uneventful portal vein resection with primary anastomosis.

**Conclusion:**

This is possibly the first documented case of portal vein renal tumor thrombosis in a case of isolated pancreatic metastasis from previously operated renal cell carcinoma in published world surgical literature.

## Background

Pancreatic metastases from previously treated renal cell carcinoma are known but uncommon. Surgical resection of pancreatic metastasis remains the only worthwhile modality of treatment. We describe our experience where a pancreatic metastasis from previously resected right sided renal cell carcinoma was resected with a subtotal left pancreatectomy with portal vein resection and primary anastomosis for a renal tumor thrombus in the splenic and portal vein.

## Case presentation

**Figure 1:**
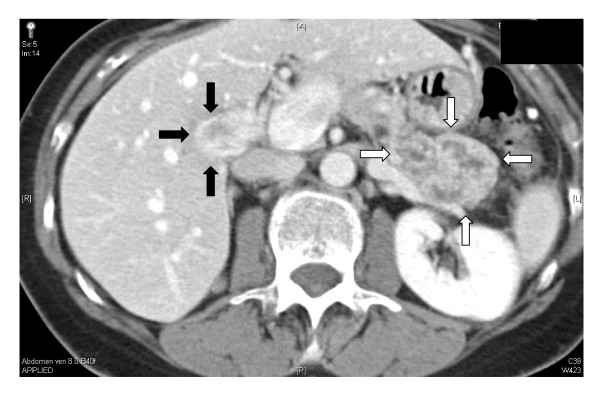
CT scan showing a pancreatic body mass and tail mass (white arrows) with portal vein thrombus (black arrows).

**Figure 2:**
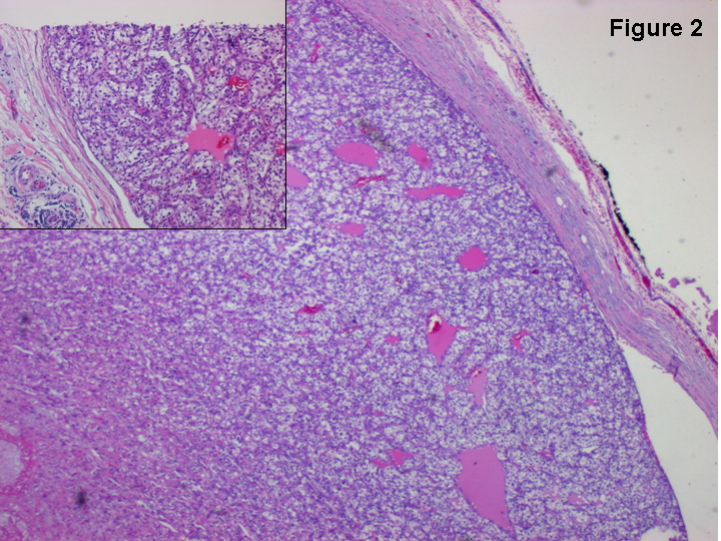
A renal cell carcinoma thrombus projects into the lumen of the portal vein and is partially attached to the venous wall (inset). (H & E staining. Original figure magnification: 50 ×; inset magnification 100 ×.)

A 62 year old patient was treated for right sided renal cell carcinoma by a radical nephrectomy in March 2004. In February 2005, he complained of abdominal pain and weight loss and on radiologic evaluation by a computed tomography (CT) scan, was diagnosed to have a pancreatic mass with evidence of portal hypertension and a portal vein thrombosis (Figure 1). In view of the solitary lesion with a differential diagnosis of primary pancreatic pathology or metastasis from renal cell carcinoma, the patient was subjected to an exploratory laparotomy in March 2005. Exploratory findings were those of a large pancreatic body and tail mass associated with signs of portal hypertension. After a careful assessment of resectability that included mobilization of the pancreatic neck from the superior mesenteric vein, a decision to perform a left sided pancreatectomy with splenectomy was taken. Division of the pancreatic neck by a vascular stapler confirmed our preoperative radiological findings. There was a large tumor thrombus in the splenic vein and it was extending via its confluence with the superior mesenteric vein into the portal vein occupying approximately 80% of its entire lumen. In view of these findings, a portal vein segment resection of 2.5 cm with primary anastomosis was added to the stapled left pancreatectomy and splenectomy. The patient had a smooth postoperative recovery and was discharged from the hospital on the 9^th ^postoperative day. Final histopathology confirmed a metastatic renal cell carcinoma to the pancreas with tumor thrombus in the portal vein (Figure 2). Furthermore, the thrombus was adherent to the wall of the vein (Figure 2, inset). The patient refused any form of adjuvant treatment and died 5 months later with systemic metastases.

## Discussion

Our case raised three questions. First, is a radial resection justified in view of a locally advanced (metastatic) pathology? Second, is a major pancreatic resection safe enough in these patients and last but not the least, the rarity of our intraoperative findings that prompted us to write this report.

Despite the unsatisfactory survival in our case, isolated pancreatic metastasis from renal call carcinoma are best treated by surgical resection. While the 5 – year survival for metastatic renal cell carcinoma is poor and hovers around 9% [1], aggressive resection of pancreatic metastasis from renal and various other tumors has shown a better survival [2-5]. Moussa *et al*., reported a mean survival of 61 months after resection for metastasis to the pancreas from renal cell carcinoma [3]. This could also be partly explained by the fact that an R0 resection of renal cell carcinoma metastasis to the pancreas is more likely than an R0 resection in primary pancreatic neoplasms and the resectability rate is around 80% [6]. Furthermore, if the disease free interval between the primary surgery and the resection of pancreatic metastasis is long, a better outcome can often be expected. In our specific case, this interval was only 11 months.

Pancreatectomy with portal vein resection is well described. A number of large studies have evaluated the safety of pancreatectomy with resection of tumor infiltrated retropancreatic vessels [7,8]. The conclusions from these studies were that segmental venous resection can be performed safely and that localized invasion of the vessel wall is not associated with a poor prognosis. Furthermore, since the results of alternative oncologic modalities continue to remain worse, it appears that aggressive pancreatic resection remains the best treatment option.

Portal hypertension in primary renal cell carcinoma is extremely rare and a medline search of recent literature revealed a solitary report of a hypernephroma infiltrating the pancreas, causing splenic vein thrombosis with resultant gastric varices as a manifestation of portal hypertension [9]. However, a medline search performed with the key words *"renal cell carcinoma, pancreatic metastasis, portal vein thrombosis" *did not reveal a single documented case of splenic and portal vein thrombosis in pancreatic metastasis from a previously treated renal cell carcinoma.

## Summary

While our group has recently published a single center experience with metastases to the pancreas from renal tumors [10], this rare clinical situation is a new recent addition and adds to the pool of existing information regarding metastatic lesions of renal cell carcinoma to the pancreas. Thus our case, probably for the first time in published literature, documents the presence of splenic and portal vein tumor thrombus in a case of pancreatic metastasis from renal cell carcinoma. Radical surgery remains the treatment mainstay and portal vein resection, especially in high volume centers, does not influence the perioperative outcome in such locally advanced surgical scenarios. While our series has documented a degree of survival benefit for metastases to the pancreas from renal cell carcinomas [10], the long-term outcome of such radical procedures needs a larger collective experience to reach a meaningful conclusion.
